# Supplementary figures and images for: The molecular basis of antigenic variation among A(H9N2) avian influenza viruses
Source: Emerg Microbes Infect. 2018 Nov 7;7:176. doi: 10.1038/s41426-018-0178-y (PMC6220119; doi:10.1038/s41426-018-0178-y)

HA1

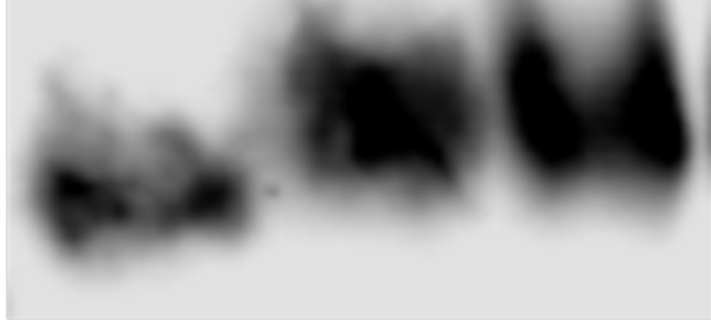

wt

T127N

L150S

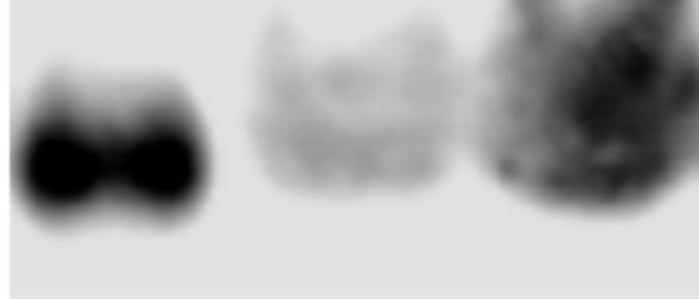

wt

T188N

D189N

Supplement: Supplementary file 1 — Figure S1 [file 41426_2018_178_MOESM1_ESM.pdf]

A

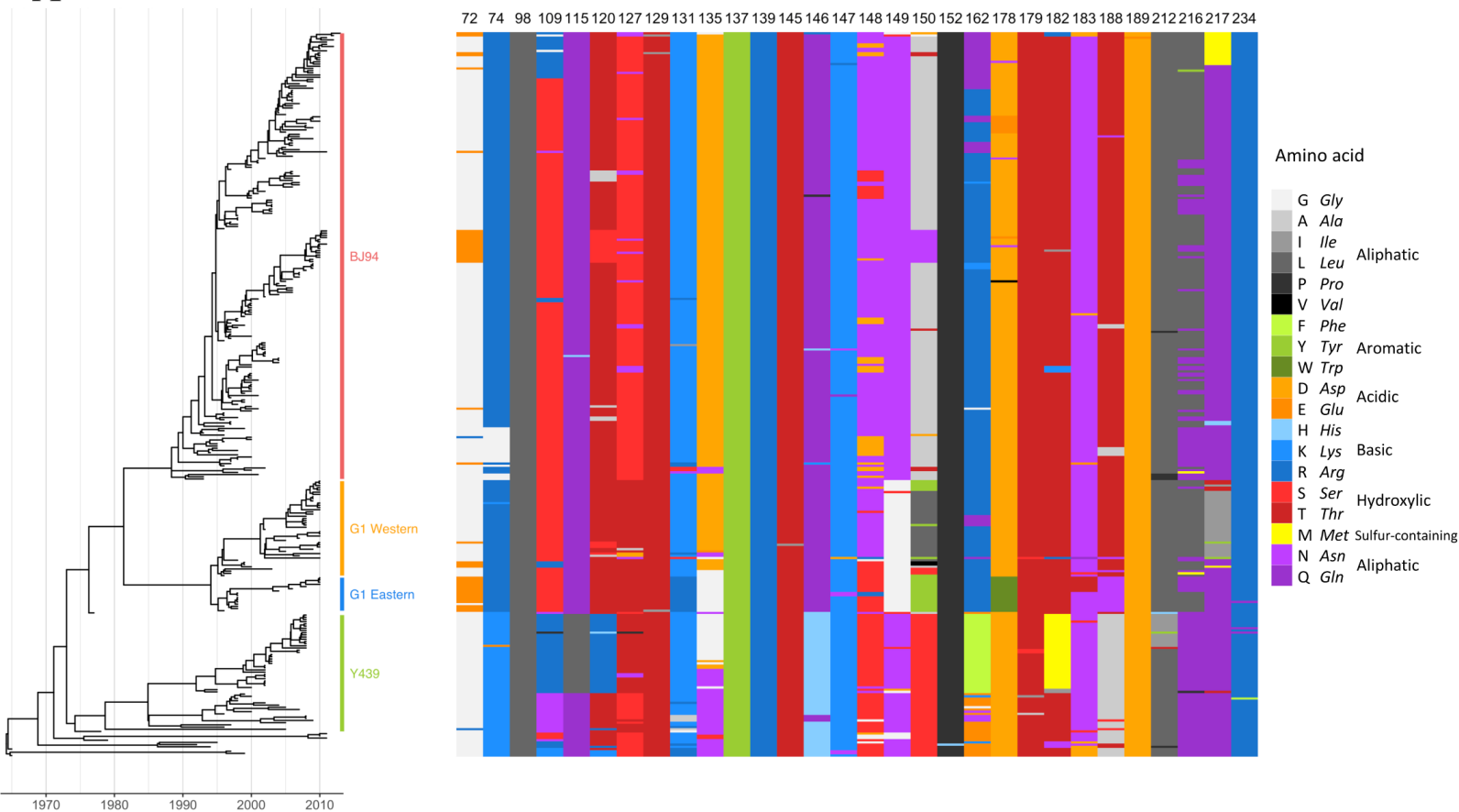

B

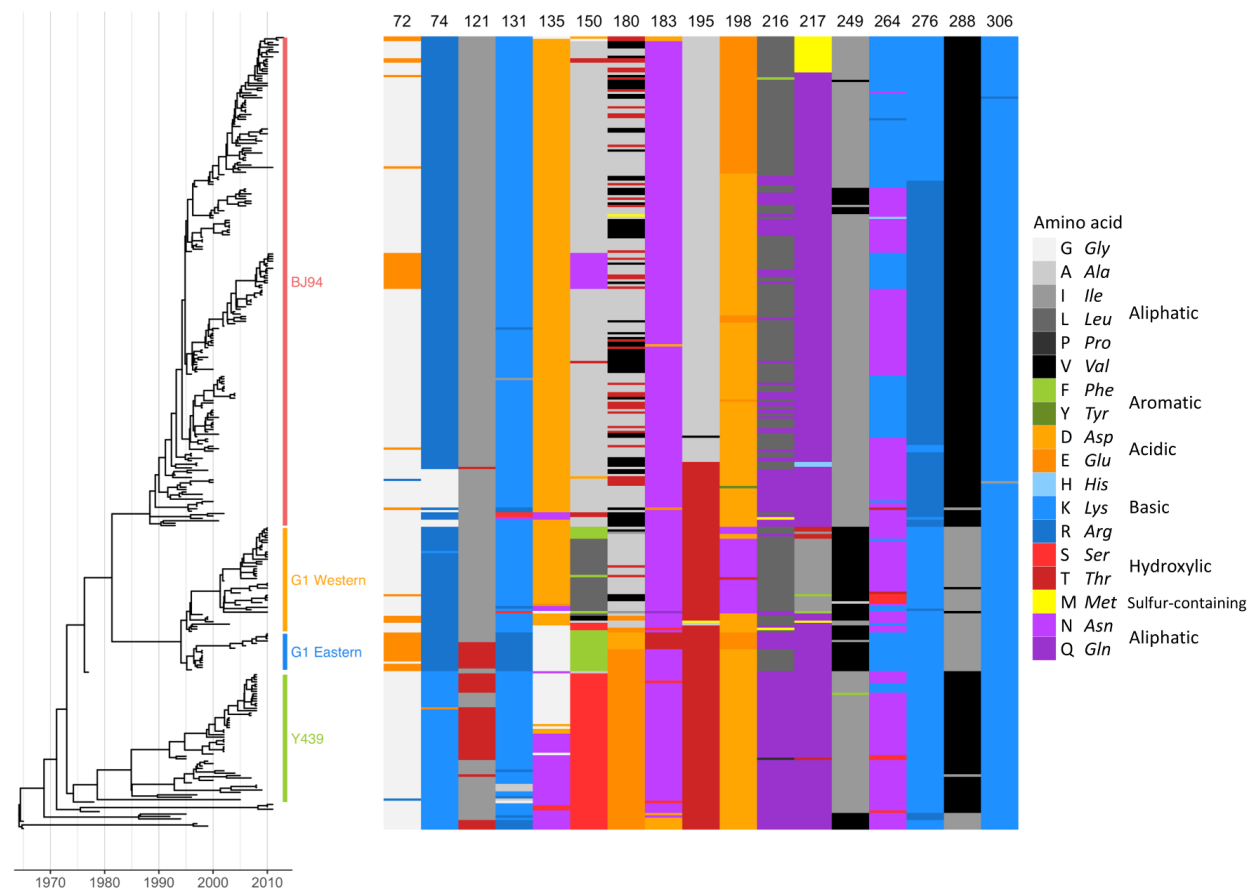

Supplement: Supplementary file 2 — Figure S2 [file 41426_2018_178_MOESM2_ESM.pdf]

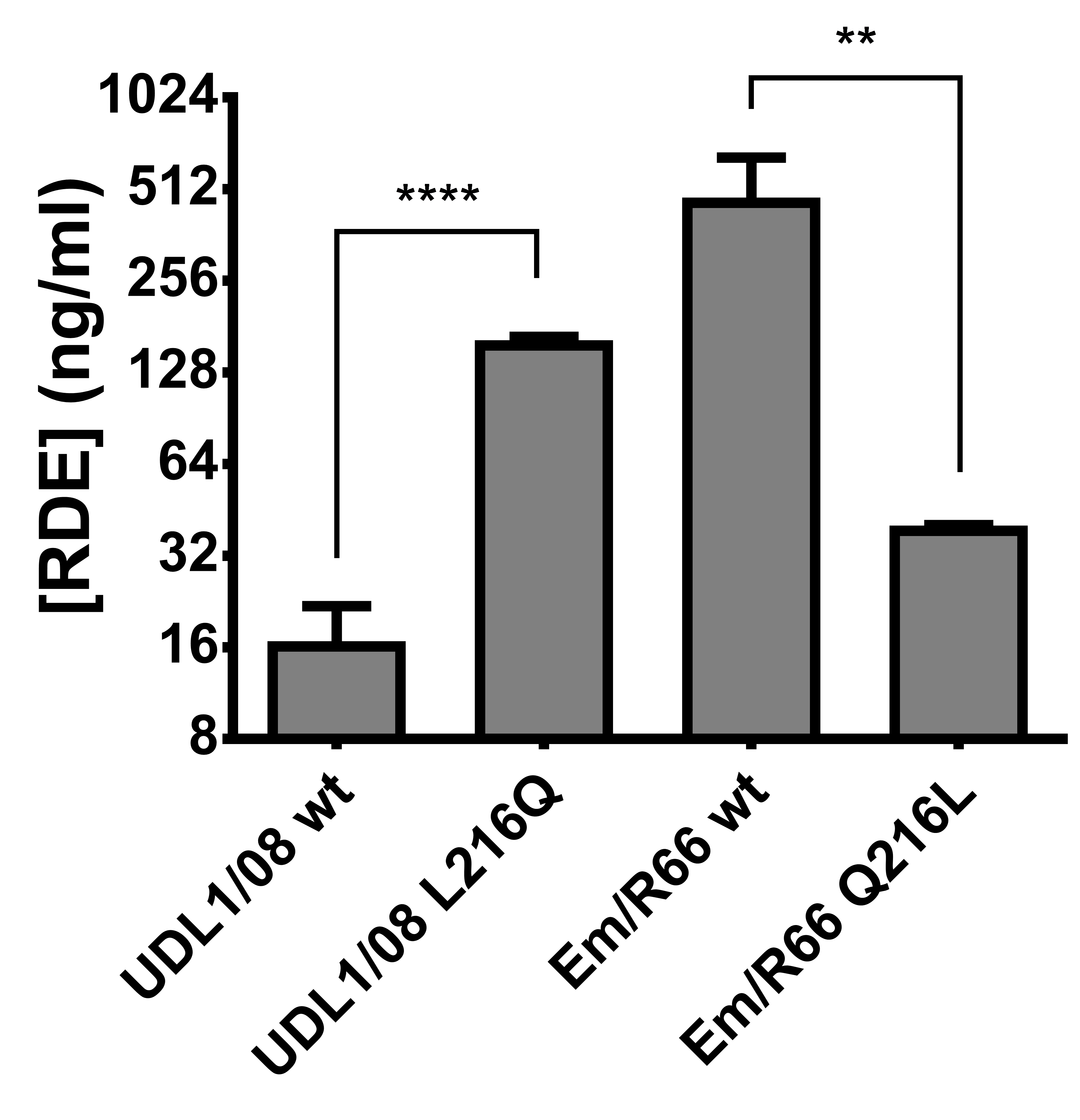

Supplement: Supplementary file 3 — Figure S3 [file 41426_2018_178_MOESM3_ESM.tif]
