# Supplementary material for: The molecular basis of antigenic variation among A(H9N2) avian influenza viruses
Source: Emerg Microbes Infect. 2018 Nov 7;7:176. doi: 10.1038/s41426-018-0178-y (PMC6220119; doi:10.1038/s41426-018-0178-y)
Supplement: Supplementary file 4 — Table S1 [file 41426_2018_178_MOESM4_ESM.pdf]

Table S1. Previously published escape mutant residues and their impact on polyclonal antisera binding.

Escape Mutation[a] (introduced mutation if different)	Antigenic site	References	Fold change in polyclonal antisera binding (log_2)[b]
G72E		Okamatsu et al, *Arch Virol* , 2008.	-0.42**
R74K (R74G)		Zhu et al, *Vet Micro* , 2015.	R74K NS, R74G -0.26*
L98Q		Okamatsu et al, *Arch Viro* l, 2008.	NS
S109I (S109R)		Kaverin et al, *JVI* , 2004.	NS
Q115P/R	H9-B	Peacock et al, *Sci Rep* , 2016.	Q115P -0.32*, Q115R -0.09 NS
T120K	H9-B	Peacock et al, *Sci Rep* , 2016.	0.00 NS
S127N[c]	Overlap	Ping et al, *Biochem biophys res comm,* 2008; Okamatsu et al, *Arch Virol* , 2008; Kaverin et al, *JVI* , 2004; Zhu et al, *Vet Micro* , 2015.	T127N[c] -2.51****, T127S -0.15 NS
T129K/A	Site I	Kaverin et al, *JVI* , 2004; Wan et al, *JVI* , 2014.	T129K -0.58****
K131N[c] (K131A/I/S)		Okamatsu et al, *Arch Virol* , 2008.	K131N[c] NR[d], K131A -0.11 NS, K131I -1.00****, K131S -0.22 NS
D135N/E (D135G)	Site II	Kaverin et al, *JVI* , 2004; Wan et al, *JVI* , 2014.	D135G -0.90****
F137L		Kaverin et al, *JVI* , 2004.	-0.47***
R139G/M	H9-B	Peacock et al, *Sci Rep* , 2016.	R139G -0.23 NS, R139M -0.03 NS
T145I	H9-A	Peacock et al, *Sci Rep* , 2016.	-1.96****
Q146K (Q146H)	Site I	Wan et al, *JVI* , 2014.	Q146K NR[d], Q146H (+T186K) -0.62****
K147T		Kaverin et al, *JVI* , 2004.	-0.02 NS
N148D	Site I	Okamatsu et al, *Arch Virol* , 2008; *Zhu et al* , Vet Micro, 2015.	-0.42**
N149D/K	Site I	Wan et al, *JVI* , 2014; Zhu et al, *Vet Micro* , 2015.	G149D -0.55****, G149K -0.92****
A150T[c]/D	Site I	Wan et al, *JVI* , 2014; Zhu et al, *Vet Micro* , 2015.	L150A +0.20 NS, L150F -0.02 NS, L150S[c] -4.81****
P152L	Site I	Kaverin et al, *JVI* , 2004.	+0.15 NS
R162W	H9-B	Peacock et al, *Sci Rep* , 2016.	-0.17 NS
D178V (D178Y)	Site II	Wan et al, *JVI* , 2014.	+0.12 NS
T179A (T179N)	Overlap	Kaverin et al, *JVI* , 2004; Zhu et al, *Vet Micro* , 2015.	-0.45***
T182I (T182R)	Site II	Okamatsu et al, *Arch Virol* , 2008; Zhu et al, *Vet Micro* , 2015.	-0.76****
N183D/S/T	Site II/H9-A	Okamatsu et al, *Arch Virol* , 2008; Kaverin et al, *JVI* , 2004; Wan et al, *JVI* , 2014; Peacock et al, *Sci Rep* , 2016.	N183D -0.72****, N183S -0.33**, N183T -0.75****
T188N[c]	Overlap	Kaverin et al, JVI, 2004.	-1.34****
D189N[c]	Site II	Wan et al, JVI, 2014.	-1.51****
L212H/P	H9-A	Okamatsu et al, *Arch Virol* , 2008; Peacock et al, *Sci Rep* , 2016.	L212P +0.25 NS
L216Q	Site II	Kaverin et al, *JVI* , 2004.	-1.51****
I217T (I217L/M/Q)	H9-A	Peacock et al, *Sci Rep* , 2016.	I217L -0.45***, I217M -0.88****, I217Q -1.32****, I217T -0.15 NS
R234Q	H9-A	Peacock et al, *Sci Rep* , 2016.	-0.13 NS

[a]H9 mature numbering used throughout.
[b]Significance determined using linear mixed models, NS = non-significant, * - P ≤ 0.05, ** - P ≤ 0.01, *** - P ≤ 0.001, **** - P ≤ 0.0001.
[c]Addition of potential glycosylation site.
[d]NR - would not rescue.
