# Supplementary material for: The molecular basis of antigenic variation among A(H9N2) avian influenza viruses
Source: Emerg Microbes Infect. 2018 Nov 7;7:176. doi: 10.1038/s41426-018-0178-y (PMC6220119; doi:10.1038/s41426-018-0178-y)
Supplement: Supplementary file 5 — Table S2 [file 41426_2018_178_MOESM5_ESM.pdf]

Table S2. Amino acid diversity and identity across different antigenic and potentially antigenic residues.

Residue	Diversity (Inverse Simpson's index)	Most common amino acid identities, >1% (%)[a]
72	1.92	**G (63.1)**[b], **E (35.1)**
74	1.29	**R (87.4), K (10.5), G (2.0)**
98	1.00	**L (99.8)**
109	2.37	**R (46.8), S (44.4)**, N (7.6)
115	1.03	**Q (98.5),** L (1.4)
120	1.26	**T (88.7),** S (6.5), R (2.5), A (1.6)
121	1.06	**I (97.1), T (2.6)**
127	2.72	**S (53.5)**, D (22.1), **T (16.4), N (6.9)**
129	1.01	**T (99.4)**
131	1.75	**K (73.9),** T (15.1), **N (3.8),** R (2.8), **S (2.0), A (1.5)**
135	2.18	**D (60.7), G (28.2)**, N (9.9)
137	1.00	**F (99.9)**
139	1.12	**R (94.5)**, K (5.3)
140	1.40	**S (82.7), N (17.3)**
145	1.01	**T (99.4)**
146	2.02	**Q (65.8),** R (23.3), H (9.4), **K (1.3)**
147	1.02	**K (99.2)**
148	1.65	**N (76.2)**, S (13.1), **D (9.3)**, G (1.0)
149	2.12	N (54.4), **G (41.7)**, S (2.7)
150	3.93	**A (41.2)**, D (23.4), **S (11.8)**, N (11.1), **L (6.0), F (1.9),** T (1.8), V (1.5)
152	1.00	**P (99.9)**
162	2.37	**Q (53.9),** R (35.4), E (7.5), **W (1.3)**
178	1.13	**D (94.0),** E (4.0), N (1.2)
179	1.06	**T (97.3), N (1.1)**
180	3.45	**A (35.6),** T (34.9), V (17.4), **E (10.8)**
182	1.73	**T (71.3), R (26.3)**
183	1.77	**N (70.2), D (26.8), S (1.2)**
186	1.35	**T (85.4), K (9.9)**, I (3.7)
188	1.29	**T (87.6)**, A (7.7), **(N 2.6)**, S (1.3)
189	1.01	**D (99.6)**
195	1.70	A (71.3), T (28.1)
198	2.32	E (57.3), **D (29.5), N (12.5)**
212	1.03	**L (98.4)**
216	1.49	**L (79.4), Q (20.1)**
217	2.59	**Q (44.6), M (41.5), I (12.4)**
234	1.02	**R (99.2)**
249	1.42	**I (82.1), V (17.6)**
264	1.85	K (67.9), N (28.0), S (2.34)
276	1.41	K (82.4), R (17.4)
288	1.44	V (81.4), I (18.4)
306	1.02	K (99.2)

[a]Diversity and amino acid identities based on total full length H9 sequences in the NCBI database as of July 2017.
[b]Mutant residues used in this study in bold.
