# Supplementary material for: The molecular basis of antigenic variation among A(H9N2) avian influenza viruses
Source: Emerg Microbes Infect. 2018 Nov 7;7:176. doi: 10.1038/s41426-018-0178-y (PMC6220119; doi:10.1038/s41426-018-0178-y)
Supplement: Supplementary file 6 — Table S3 [file 41426_2018_178_MOESM6_ESM.pdf]

Table S3. Table of viruses unable to be rescued, required additional mutations to rescue, or that reverted or gained compensatory mutations upon rescue.

Attempted mutant	Rescued?	Compensatory change	Additional mutation needed	Reverted
UDL1/08 K131N[a]	No	n/a	n/a	n/a
UDL1/08 D135G	yes	n/a	n/a	Yes, partial[c]
HK33982 G135D	yes	n/a	n/a	Yes, partial
UDL1/08 Q146K	no	n/a	n/a	n/a
UDL1/08 Q146H	no	n/a	Yes, T186K	n/a
UDL1/08 A180E	no	n/a	Yes, L216Q, I217I or I217Q	n/a
UDL1/08 A180D	yes	n/a	n/a	Yes, D180G (mixed, equal)
HK33982 D180A	yes	Yes, G215D (total change[b])	n/a	n/a
UDL1/08 T186K	no	n/a	n/a	n/a
R66 N198D	no	n/a	n/a	n/a
HK33982 Q216L	yes	Yes, H174E (total change)	n/a	n/a

[a]Indicates addition of potential glycosylation site.

[b]'Total change' indicates presence of singlet on.

[c]'Partial' indicates doublet with no change to consensus.
