# Supplementary material for: The molecular basis of antigenic variation among A(H9N2) avian influenza viruses
Source: Emerg Microbes Infect. 2018 Nov 7;7:176. doi: 10.1038/s41426-018-0178-y (PMC6220119; doi:10.1038/s41426-018-0178-y)
Supplement: Supplementary file 7 — Table S4 [file 41426_2018_178_MOESM7_ESM.pdf]

Table S4. Literature used for haemagglutination inhibition assay/phylogenetics modelling input.

Reference	No. of Antisera/ Virus pairs
Wei, Y. *et al.* Antigenic evolution of H9N2 chicken influenza viruses isolated in China during 2009-2013 and selection of a candidate vaccine strain with broad cross-reactivity. *Vet Microbiol* **182**, 1-7 (2016)	330
Gerloff, N. A. *et al.* Genetically Diverse Low Pathogenicity Avian Influenza A Virus Subtypes Co-Circulate among Poultry in Bangladesh. *PLoS One* **11**, e0152131 (2016)	293
Zhang, Y. *et al.* Molecular and antigenic characterization of H9N2 avian influenza virus isolates from chicken flocks between 1998 and 2007 in China. *Vet Microbiol* **156**, 285-293 (2012)	264
Wang, Y., Davidson, I., Fouchier, R. & Spackman, E. Antigenic Cartography of H9 Avian Influenza Virus and its Application to Vaccine Selection. *Avian diseases* **60**, 218-225 (2015)	264
Sun, Y. *et al.* Genotypic evolution and antigenic drift of H9N2 influenza viruses in China from 1994 to 2008. *Vet Microbiol* **146**, 215-225 (2010)	253
Shanmuganatham, K. *et al.* Antigenic and Molecular Characterization of Avian Influenza A(H9N2) Viruses, Bangladesh. *Emerg Infect Dis* **19**, 1393-1402 (2013)	203
Guan, Y. *Continuing evolution of H9N2 avian influenza A viruses in poultry in southern China* Master of Philosophy thesis, Hong Kong University, (2011)	192
Nomura, N., Sakoda, Y., Soda, K., Okamatsu, M. & Kida, H. An H9N2 influenza virus vaccine prepared from a non-pathogenic isolate from a migratory duck confers protective immunity in mice against	133
challenge with an H9N2 virus isolated from a girl in Hong Kong. *J Vet Med Sci* **74**, 441-447 (2012)	
SJCEIRS H9N2 Working Group. Assessing the fitness of distinct clades of influenza A (H9N2) viruses. *Emerg Microbes Infect* **2**, e75 (2013)	132
Li, C. et al. Evolution of H9N2 influenza viruses from domestic poultry in Mainland China. *Virology* **340**, 70-83 (2005)	126
Xue, Y. *et al.* Sequence and phylogenetic analysis of surface protein genes of emerging H9N2 influenza viruses isolated from poultry in two geographical regions of China. *Virus Genes* **48**, 479-485 (2014)	108
Lin, Y. P. *et al.* Avian-to-human transmission of H9N2 subtype influenza A viruses: relationship between H9N2 and H5N1 human isolates. *Proc Natl Acad Sci USA* **97**, 9654-9658 (2000).	90
Guan, Y., Shortridge, K. F., Krauss, S. & Webster, R. G. Molecular characterization of H9N2 influenza viruses: were they the donors of the "internal" genes of H5N1 viruses in Hong Kong? *Proc Natl Acad Sci USA* **96**, 9363-9367 (1999)	90
Shen, H. Q. *et al.* Isolation and phylogenetic analysis of hemagglutinin gene of H9N2 influenza viruses from chickens in South China from 2012 to 2013. *J Vet Sci* **16**, 317-324 (2015)	81
Liu, J. *et al.* H9N2 influenza viruses prevalent in poultry in China are phylogenetically distinct from A/quail/Hong Kong/G1/97 presumed to be the donor of the internal protein genes of the H5N1 Hong Kong/97 virus. *Avian Pathol* **32**, 551-560(2003)	70
Zhu, Y. *et al.* Phylogenetic and antigenic characterization of reassortant H9N2 avian influenza viruses isolated from wild waterfowl in the East Dongting Lake wetland in 2011-2012. *Virol J* **11**, 77, (2014)	64
Nguyen, D. C. *et al.* Isolation and characterization of avian influenza viruses, including highly pathogenic H5N1, from poultry in live bird markets in Hanoi, Vietnam, in 2001. *J Virol* **79**, 4201-4212 (2005)	63
Mase, M., Eto, M., Imai, K., Tsukamoto, K. & Yamaguchi, S. Characterization of H9N2 influenza A viruses isolated from chicken products imported into Japan from China. *Epidemiol Infect* **135**, 386-391 (2007)	56
Huang, Y. *et al.* Human infection with an avian influenza A (H9N2) virus in the middle region of China. *J Med Virol* **87**, 1641–1648 (2015)	56
Guan, Y. *et al.* H9N2 influenza viruses possessing H5N1-like internal genomes continue to circulate in poultry in southeastern China. *J Virol* **74**, 9372-9380 (2000)	54
Nomura, N. *et al.* Characterization of avian influenza viruses isolated from domestic ducks in Vietnam in 2009 and 2010. *Arch Virol* **157**, 247-257 (2012)	54
Pu, J. *et al.* Evolution of the H9N2 influenza genotype that facilitated the genesis of the novel H7N9 virus. *Proc Natl Acad Sci USA* **112**, 548-553 (2015)	50
Choi, Y. K., Seo, S. H., Kim, J. A., Webby, R. J. & Webster, R. G. Avian influenza viruses in Korean live poultry markets and their pathogenic potential. *Virology* **332**, 529-537 (2005)	48
Cameron, K. R. *et al.* H9N2 subtype influenza A viruses in poultry in pakistan are closely related to the H9N2 viruses responsible for human infection in Hong Kong. *Virology* **278**, 36-41 (2000)	48
Cong, Y. L. *et al.* Swine infection with H9N2 influenza viruses in China in 2004. *Virus Genes* **36**, 461-469 (2008)	48
Cong, Y. L. *et al.* Antigenic and genetic characterization of H9N2 swine influenza viruses in China. *J Gen Virol* **88**, 2035-41 (2007)	40
Negovetich, N. J. *et al.* Live bird markets of Bangladesh: H9N2 viruses and the near absence of highly pathogenic H5N1 influenza. *PLoS One* **6**, e19311 (2011)	40
Kandeil, A. *et al.* Genetic and antigenic evolution of H9N2 avian influenza viruses circulating in Egypt between 2011 and 2013. *Arch Virol* **159**, 2861-2876 (2014)	38
Wernery, U. *et al.* H9N2 influenza viruses from birds used in falconry. *Influenza Other Respir Viruses* **7**, 1241-45 (2013)	35
Saito, T. *et al.* Characterization of a human H9N2 influenza virus isolated in Hong Kong. *Vaccine* **20**, 125-133 (2001)	32
Okamatsu, M. *et al.* The genetic and antigenic diversity of avian influenza viruses isolated from domestic ducks, muscovy ducks, and chickens in northern and southern Vietnam, 2010-2012. *Virus Genes* **47**, 317-329 (2013)	32
Zhang, H., Xu, B., Chen, Q. & Chen, Z. Characterization of H9N2 influenza viruses isolated from Dongting Lake wetland in 2007. *Arch Virol* **156**, 95-105 (2011)	32
Zhang, P. *et al.* Characterization of H9N2 influenza viruses isolated from vaccinated flocks in an integrated broiler chicken operation in eastern China during a 5 year period (1998-2002). *J Gen Virol* **89**, 3102-3112 (2008)	26
Webster, R. G. & Hulse, D. J. Microbial adaptation and change: avian influenza. *Revue scientifique et technique* **23**, 453-465 (2004).	25
Kim, J. A., Cho, S. H., Kim, H. S. & Seo, S. H. H9N2 influenza viruses isolated from poultry in Korean live bird markets continuously evolve and cause the severe clinical signs in layers. *Vet Microbiol* **118**, 169-176 (2006)	24
Sorrell, E. M., Wan, H., Araya, Y., Song, H. & Perez, D. R. Minimal molecular constraints for respiratory droplet transmission of an avian-human H9N2 influenza A virus. *Proc Natl Acad Sci USA* **106**, 7565-7570 (2009)	20
Park, K. J. *et al.* Rapid evolution of low-pathogenic H9N2 avian influenza viruses following poultry vaccination programmes. *J Gen Virol* **92**, 36-50 (2011)	19
World Health Organisation. Antigenic and genetic characteristics of influenza A(H5N1) and influenza A(H9N2) viruses and candidate vaccine viruses developed for potential use in human vaccines *WHO Global Influenza Programme* **February 2010** (2010)	18
Peiris, J. S. *et al.* Cocirculation of avian H9N2 and contemporary "human" H3N2 influenza A viruses in pigs in southeastern China: potential for genetic reassortment? *J Virol* **75**, 9679-9686 (2001)	18
Dong, G. *et al.* Reassortant H9N2 influenza viruses containing H5N1-like PB1 genes isolated from black-billed magpies in Southern China. *PLoS One* **6**, e25808 (2011)	18
Aamir, U. B., Wernery, U., Ilyushina, N. & Webster, R. G. Characterization of avian H9N2 influenza viruses from United Arab Emirates 2000 to 2003. *Virology* **361**, 45-55 (2007)	15
World Health Organisation. Antigenic and genetic characteristics of zoonotic influenza viruses and development of candidate vaccine viruses for pandemic preparedness *WHO Global Influenza Programme* **February 2014** (2014)	15
Wang, B. *et al.* Genotype diversity of H9N2 viruses isolated from wild birds and chickens in Hunan Province, China. *PLoS One* **9**, e101287 (2014)	12
Peiris, M. *et al.* Human infection with influenza H9N2. *Lancet* **354**, 916-917 (1999)	11
Moon, H. J. *et al*. Active reassortment of H9 influenza viruses between wild birds and live-poultry markets in Korea. *Arch Virol* **155**, 229-41 (2010)	9
Lee, Y. J. *et al.* Continuing evolution of H9 influenza viruses in Korean poultry. *Virology* **359**, 313-323 (2007)	9
Peiris, M., Yam, W. C., Chan, K. H., Ghose, P. & Shortridge, K. F. Influenza A H9N2: aspects of laboratory diagnosis. *J Clin Microbiol* **37**, 3426-3427 (1999)	6
Bahari, P., Pourbakhsh, S. A., Shoushtari, H. & Bahmaninejad, M. A. Molecular characterization of H9N2 avian influenza viruses isolated from vaccinated broiler chickens in northeast Iran. *Tropic Anim Health Prod* **47**, 1195-1201 (2015)	5
Previously unpublished (see Supplementary Table S5)	272
Total	3941
