# Supplementary material for: The molecular basis of antigenic variation among A(H9N2) avian influenza viruses
Source: Emerg Microbes Infect. 2018 Nov 7;7:176. doi: 10.1038/s41426-018-0178-y (PMC6220119; doi:10.1038/s41426-018-0178-y)
Supplement: Supplementary file 8 — Table S5 [file 41426_2018_178_MOESM8_ESM.pdf]

Table S5. Additional, previously unpublished haemagglutination inhibition data used in modelling analysis.

Virus	Antisera (species antisera raised in)															
	A/chicken/Pakistan/UDL-01/2008 (chicken)	A/chicken/Pakistan/UDL-02/2008 (chicken)	A/chicken/Saudi Arabia/D-36363/2010 (ferret	A/Bangladesh/0994/2011 (ferret)	A/chicken/Iran/B102/2005 (chicken)	A/chicken/Lebanon/1080/2004 (chicken)	A/chicken/Emirates/R66/2002 (chicken)	A/quail/Dubai/302/2000 (ferret)	A/chicken/Pakistan/2/1999 (chicken)	A/quail/Hong Kong/G1/1997 (chicken)	A/Hong Kong/33982/2009 (chicken)	A/Hong Kong/1073/1999 (sheep)	A/mallard/England/7798-6499/2006 (ferret)	A/knot/England/497/2002 (chicken)	A/duck/Hong Kong/91/1976 (chicken)	A/turkey/Wisconsin/1/1966 (chicken)
A/chicken/Pakistan/UDL-01/2008	**8192**[a]	512	4096	2048	4096	2048	512	2048	512	128	64	1024	128	32	512	<64[b]
A/chicken/Pakistan/UDL-02/2008	4096	**1024**	2048	2048	1024	1024	256	1024	512	64	32	1024	256	64	512	128
A/environment/Bangladesh/10306/2011	512	64	64	512	256	256	16	64	128	32	32	256	16	<8	64	<64
A/Bangladesh/0994/2011	2048	256	512	**512**	1024	512	128	256	256	64	32	512	64	32	256	<64
A/quail/United Arab Emirates/D1556/2011	128	64	64	128	32	16	16	8	<32	64	16	256	32	<8	64	<64
A/chicken/Egypt/D7100/2013	8192	256	512	128	1024	2048	256	512	512	64	64	1024	256	64	512	128
A/chicken/India/WB-NIV1057169/2010	4096	512	2048	4096	2048	2048	256	1024	512	128	64	1024	128	32	512	128
A/chicken/Israel/239/2013	8192	512	1024	512	2048	2048	256	1024	512	128	64	1024	128	64	512	<64
A/chicken/Lebanon/1080/2004	4096	256	512	2048	2048	**8192**	1024	512	512	256	64	1024	128	64	512	<64
A/chicken/Emirates/R66/2002	128	64	32	16	64	128	**256**	256	128	64	16	256	32	32	128	<64
A/quail/Hong Kong/G1/1997	256	32	16	2048	128	128	256	128	128	**512**	128	1024	16	32	256	<64
A/Hong Kong/33982/2009	128	32	16	1024	128	64	32	128	64	64	**1024**	256	4	<8	128	128
A/Chinese hwamei/Vietnam/38/06	64	32	16	1024	64	64	32	64	64	32	512	256	8	<8	128	128
A/chicken/Hong Kong/G9/1997	2048	128	256	128	1024	1024	128	256	256	64	32	1024	128	32	256	<64
A/chicken/Wenzhou/606/2013	128	256	64	2048	64	128	32	64	64	64	128	512	64	64	256	<64
A/Hong Kong/3239/2008	4096	256	256	128	1024	1024	128	256	256	64	32	1024	64	32	256	<64
A/turkey/Wisconsin/1/1966	512	128	16	16	32	32	32	64	128	128	256	256	512	64	1024	**512**

[a]Homologous titres shown in bold.

[b]< indicates no detectable cross-reactivity could be found at range of concentrations used.
