# Supplementary material for: The molecular basis of antigenic variation among A(H9N2) avian influenza viruses
Source: Emerg Microbes Infect. 2018 Nov 7;7:176. doi: 10.1038/s41426-018-0178-y (PMC6220119; doi:10.1038/s41426-018-0178-y)
Supplement: Supplementary file 9 — Table S6 [file 41426_2018_178_MOESM9_ESM.pdf]

Table S6. Impact on HI titres of substitutions introduced by reverse genetics at positions identified by modelling.

Position[a]	Substitution	Background	Change in titre (log_2)			
			vs. parent-like antisera[b]	vs. mutant-like antisera[b]	Avidity impact[c]	Antigenic effect[c]
Reciprocal mutations						
72	G72E	UDL1/08	-0.42**	+0.44**	NS	0.64
	E72G	HK33982	-0.34*	+1.38****	NS	0.64
121	I121T	UDL1/08	-0.46***	+0.10 NS	-0.22	NS
	T121I	HK33982	+0.72****	+0.66****	0.22	NS
135	D135G	UDL1/08	-0.90****	-0.16 NS	-0.41	0.51
	G135D	HK33982	-0.13 NS	+0.73****	0.41	0.51
150	L150A	UDL1/08	+0.20 NS	+0.63***	0.46	0.32
	A150L	Em/R66	-0.83****	-0.05 NS	-0.46	0.32
	L150F	UDL1/08	-0.02 NS	+0.68****	0.27	0.36
	F150L	HK33982	+0.07 NS	+1.07****	-	-
180	A180E	UDL1/08 (+L216Q/I217L)	-0.61****	+0.65****	-0.25	0.86
	E180A	Em/R66	-0.54****	+1.51****	0.25	0.86
183	N183T	UDL1/08	-0.75****	+0.42**	NS	1.06
	T183N	HK33982	-0.90****	-0.04 NS	NS	1.06
216	L216Q	UDL1/08	-1.54****	-1.63****	-1.68	0.29
	Q216L	Em/R66	+0.83****	+1.83****	1.68	0.29
217	I217L	UDL1/08	-0.45***	-1.11****	-0.22	NS
	L217I	Em/R66	+0.09 NS	+0.06 NS	0.22	NS
	I217Q	UDL1/08	-1.32****	-0.22 NS	-0.74	NS
	Q217I	HK33982	+1.19****	+1.08****	0.74	NS
249	I249V	Em/R66	-1.09****	-0.83****	-0.77	NS
	V249I	UDL1/08	+0.79****	+0.18 NS	0.77	NS
Non reciprocal mutations						
74	R74G	UDL1/08	-0.26*			
131	K131A	UDL1/08	-0.11 NS			
	K131I	UDL1/08	1.00****			
	K131S	UDL1/08	-0.22 NS			
183	N183D	UDL1/08	-0.72****			
198	D198N	UDL1/08	+0.27 NS			

[a]H9 mature numbering used throughout.

[b]Significance determined using linear mixed models; NS = non-significant, *= <0.05, **= <0.01, ***= <0.001, ****= <0.0001.

[c]Avidity and antigenic impacts of substitutions estimated using Equation 1. NS indicates a model lacking the relevant effect was favoured by DIC
