# Supplementary material for: The molecular basis of antigenic variation among A(H9N2) avian influenza viruses
Source: Emerg Microbes Infect. 2018 Nov 7;7:176. doi: 10.1038/s41426-018-0178-y (PMC6220119; doi:10.1038/s41426-018-0178-y)
Supplement: Supplementary file 10 — Table S7 [file 41426_2018_178_MOESM10_ESM.pdf]

Table S7. Effect of mutations used in this study on monoclonal antibody binding.

UDL1/08 mutant	Reduction in HI titre by escape mutant (Log2)							Suggested antigenic site
	H9-A binding mAbs				H9-B binding mAbs			
	CG12	EC12	HA9	JF7	IB3	ID2	JF8	
Wild-type	-[a]	-	-	-	-	-	-	
G72E	-	-	-	-	2	-	-	H9-B
R74G	-	-	-	-	-	-	-	
R74K	-	-	-	-	-	-	-	
L98Q	-	-	-	-	-	-	-	
S109R	-	-	-	-	-	-	-	
I121T	-	-	-	-	-	-	-	
T127S	-	-	-	-	-	-	-	
T127N[b]	2	2	2	3	2	3	3	Glycosylation[c]
T129K	-	-	-	-	-	-	-	
K131A	-	-	-	-	-	-	-	
K131I	1	-	1	2	2	1	2	Avidity?[d]
K131S	-	-	-	-	-	-	-	
D135G	-	-	-	-	-	2	1	H9-B
F137L	-	-	-	-	-	-	-	
K147T	-	-	-	-	-	-	-	
N148D	-	-	-	-	-	-	-	
G149D	-	-	-	-	-	-	-	
G149K	3	4	3	3	-	-	-	H9-A
L150A	-	-	-	-	-	-	-	
L150F	-	-	-	-	-	-	-	
L150S[b]	3	3	3	4	>4[e]	>4	>4	Glycosylation
P152L	-	-	-	-	-	-	-	
D178Y	-	1	1	-	1	2	1	Avidity?
T179N	-	-	-	2	-	-	-	H9-A
A180D	>4	>3	>3	>4	-	-	-	H9-A
A180E + L216Q	-	-	-	3	-	-	-	H9-A
T182R	1	-	-	-	-	1	1	Avidity?
T188N[b]	1	1	1	2	1	2	2	Glycosylation
D189N[b]	1	1	1	2	1	2	2	Glycosylation
N198D	-	-	-	-	-	-	-	
L216Q	1	1	1	2	1	3	3	Avidity?
I217L	-	-	1	-	-	-	-	H9-A?
I217M	1	1	1	1	1	2	2	Avidity?
I217Q	1	1	1	-	-	2	2	Avidity?
V249I	-	-	-	-	-	-	-	
Mutants in an Em/R66 background								
Wild-type Em/R66	2	2	2	>4	>4	>4	>4	
E180A	2	2	2	2	>4	>4	>4	H9-A
Q216L	-	-	-	>4	>4	1	>4	Avidity?
L217I	2	2	2	>4	>4	>4	>4	
Mutants in an HK/33982 background								
Wild-type HK/33982	>5	>5	>5	>5	>5	>5	>5	
E72G	>5	>5	>5	>5	>5	>5	>5	
G135D	>5	>5	>5	>5	>5	3	>5	H9-B
H174E	>5	>5	>5	>5	>5	2	>5	Avidity?
T183N	>5	>5	>5	>5	>5	>5	>5	
L216Q +H174E	>5	>5	>5	>5	>5	>5	>5	
Q217I	>5	>5	>5	>5	>5	3	>5	Avidity?

[a]'-' indicates no difference from wild-type UDL1/08.

[b]Potential glycosylation site mutants.

[c]Mutant likely modulates binding from both groups of mAbs due to adding a glycosylation site.

[d]Non-glycosylation site mutants that modulate reactivies of antibodies from both H9-A and H9-B are considered potential avidity mutations.

[e]> indicates antibodies unable to inhibit mutant virus at the highest concentration tested.
